# The Association of Carbohydrate Antigen (CA) 19-9 Levels and Low Skeletal Muscle Mass in Healthy Adults

**DOI:** 10.3390/nu15153394

**Published:** 2023-07-30

**Authors:** Jae Hyun Lee, Dong-Kun Kim, Mi-Yeon Lee, Han-Sol Lim, Min-Jung Kwon, Yong-Taek Lee, Kyung Jae Yoon, Chul-Hyun Park

**Affiliations:** 1 Department of Rehabilitation Medicine, Kosin University College of Medicine, Busan 49267, Republic of Korea; sense0906@gmail.com; 2 Department of Artificial Intelligence Convergence, Pukyong National University, Busan 48513, Republic of Korea; 3 Department of Physical and Rehabilitation Medicine, Kangbuk Samsung Hospital, Sungkyunkwan University School of Medicine, 29 Saemunan-ro, Jongno-gu, Seoul 03181, Republic of Korea; dongkun717@gmail.com (D.-K.K.); hslim0622@gmail.com (H.-S.L.); yongtaek1.lee@gmail.com (Y.-T.L.); kint99@gmail.com (K.J.Y.); 4 Division of Biostatistics, Department of R&D Management, Kangbuk Samsung Hospital, Sungkyunkwan University School of Medicine, Seoul 03181, Republic of Korea; my7713.lee@samsung.com; 5 Department of Laboratory Medicine, Kangbuk Samsung Hospital, Sungkyunkwan University School of Medicine, Seoul 03181, Republic of Korea; mjk.kwon@samsung.com

**Keywords:** carbohydrate antigen 19-9, skeletal muscle mass, population study

## Abstract

Carbohydrate antigen 19-9 (CA 19-9) is a commonly used tumor marker for pancreatic cancer. However, CA 19-9 can be overexpressed in several benign inflammatory diseases. We investigated the relationship between high CA 19-9 level and low muscle mass (LMM) in healthy adults without cancer. Participants who underwent evaluation of muscle mass and CA 19-9 were included. Exclusion criteria were any malignancy, cardiovascular disease, tuberculosis, and chronic lung/liver disease. Participants were classified into “normal”, “mild LMM”, and “severe LMM” groups based on the skeletal muscle mass index. Multivariable logistic regression analyses were conducted to assess the association of high CA 19-9 with muscle mass status. A total of 263,061 adults were included. The mean age and SMI were 41.03 years and 7.13 kg/m^2^. After adjustments for various confounders, high CA 19-9 was independently associated with mild LMM (adjusted odds ratio, 1.677 [95% confidence interval, 1.533–1.834]) and severe LMM (2.651 [2.126–3.306]) compared to the normal group. Furthermore, the association between high CA 19-9 and severe LMM was stronger in men than in women. Elevated CA 19-9 levels were independently associated with a higher prevalence of LMM in healthy adults without cancer. Therefore, increased CA 19-9 could be utilized as a novel biomarker for sarcopenia.

## 1. Introduction

Skeletal muscle plays a vital role in the regulation of homeostasis and functions in various organs, and altered body composition with lower muscle mass (LMM) and increased adipose tissue can enhance proinflammatory conditions, insulin resistance, and oxidative stress [1,2]. LMM has recently gained attention due to its negative prognostic implications. It is also a critical indicator of poor prognosis and increased risk of various diseases and overall mortality [3,4]. Thus, research on risk factors and biomarkers related to LMM is actively being conducted [5,6]. Many well-established conditions from comorbidities to demographic factors, such as age, inactivity, and malnutrition, have been correlated with LMM. However, even younger and physically active individuals could have LMM [7].

Carbohydrate antigen 19-9 (CA 19-9) is widely used to aid diagnosis and to assess the prognosis and chemotherapy response of pancreatic cancer [8,9,10]. CA 19-9 is a tetrasaccharide carbohydrate referred to as sialyl Lewis a, which is part of the Lewis family of blood group antigens. It is synthesized by exocrine epithelial cells, with its primary source being human pancreatic and biliary ductal cells [8]. While CA 19-9 is typically present in small quantities in the serum, overexpression occurs in cases of malignant transformation of epithelial cells [9,10]. It has also been reported that inflammatory conditions involve these epithelial cells, and even benign conditions unrelated to pancreatobiliary diseases can lead to high levels of CA 19-9 [11,12,13,14]. Recently, some studies have reported sustained elevation of CA 19-9 in the absence of definite causative conditions [15,16].

LMM is a common characteristic of cancer, and in the context of cancer cachexia, elevation of CA 19-9 may co-occur with LMM as the disease progresses. However, it is currently unclear whether there is an independent correlation between these two variables. Furthermore, because there are several unknown causes of CA 19-9 elevation in the general population, it is possible that LMM and CA 19-9 are correlated in the general population. To date, no study has investigated this potential relationship in a healthy adult population without cancer. Therefore, our study aimed to investigate the possible link between CA 19-9 levels and LMM among apparently healthy adults.

## 2. Materials and Methods

### 2.1. Study Population

We recruited 379,206 adults aged from 18 to 89 years who underwent evaluations of both the CA 19-9 level and body composition analysis as a comprehensive annual or biennial health checkup program in Kangbuk Samsung Hospital Total Healthcare Centers in Seoul and Suwon, Republic of Korea, between 1 January 2012 and 31 December 2019.

**Figure 1:**
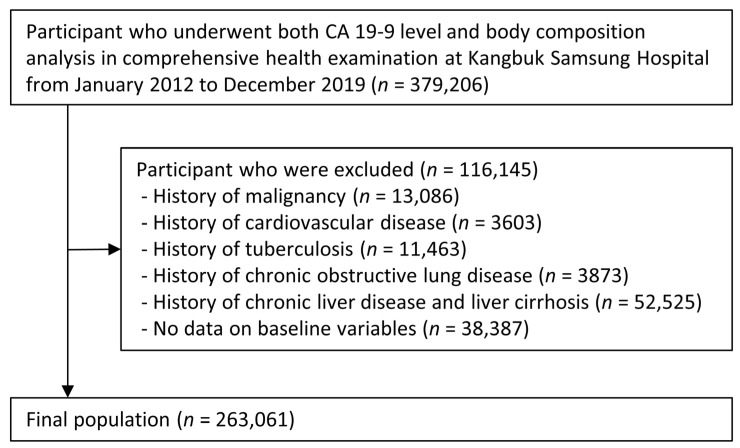
Selection of study population. CA 19-9: carbohydrate antigen 19-9.

The participants were excluded based on the following exclusion criteria: history of any malignancy (*n* = 13,086), history of chronic obstructive pulmonary disease (*n* = 3873), history of tuberculosis (*n* = 11,463), history of cardiovascular disease (*n* = 3603), history of chronic liver disease or liver cirrhosis (*n* = 52,525), and participants with missing baseline variables (*n* = 38,387). Some individuals met more than one exclusion criteria. After the exclusion (*n* = 116,145), a total of 263,061 participants were included in the final analysis (Figure 1).

The Kangbuk Samsung Hospital’s Institutional Review Board (IRB) gave its approval to our study protocol (IRB no. KBSMC 2023-05-055). We used de-identified datasets typically gathered as part of the health screening exam. Thus, the IRB exempted us from the need for informed consent. Our study was performed in accordance with the ethical standards laid down in the 1964 Declaration of Helsinki and its later amendments.

### 2.2. Measurements

All the data for the study were collected during the comprehensive health examination of the study participants. Participants were examined after at least 10 h of fasting and completed the standardized questionnaire form that provides information about demographic characteristics, past medical history, and behavior history including smoking status, alcohol drinking, and physical activity.

Blood samples for measurement of serum CA 19-9, triglycerides, total cholesterol, low-density lipoprotein cholesterol (LDL), high-density lipoprotein cholesterol (HDL), fasting insulin, fasting glucose, aspartate transaminase (AST), alanine aminotransferase (ALT), alkaline phosphatase, total bilirubin, creatinine, and *C*-reactive protein (CRP) were collected. Serum CA 19-9 was determined using an electrochemiluminescence immunoassay (Modular E170; Roche Diagnostics, Tokyo, Japan) from January 2012 to April 2015 and using the cobas 8000 e602 (Roche Diagnostics, Indianapolis, IN, USA) until February 2018. From that point on, the measurement was performed using the cobas 8000 e802 (Roche Diagnostics). From the Korean Society of Laboratory Medicine and Korean Association of Quality Assurance for Clinical Laboratories, Kangbuk Samsung Hospital’s Laboratory Medicine Department has received accreditation [17]. Additionally, the laboratory participates in the College of American Pathologists’ survey proficiency testing program.

Trained medical personnel measured the blood pressure (BP) and performed the following anthropometric measurements: height (cm) and body mass (kg). BP was measured using an automated oscillometric device (53,000, Welch Allyn, New York, NY, USA) while participants were in a sitting position with the arm supported at the heart level. Hypertension was defined as systolic BP (SBP) ≥ 140 mm Hg, diastolic BP ≥ 90 mm Hg, or the use of antihypertensive medications. BMI (body mass index) was calculated by dividing body mass by square of height (m^2^). There were three categories for smoking status: current smoker, former smoker, and non-smoker. Those who were smoking at the time of the health examination and had smoked more than 100 cigarettes during their lifetime were considered current smokers. Former smokers were those who had smoked more than >100 cigarettes during their lifetime but were not smoking at the time of the interview. Non-smokers were defined as those who did not match both criteria [18]. Heavy alcohol drinking was defined as the intake of more than >20 g of ethanol/day [19,20]. The physical activity-related energy expenditure, quantified as metabolic equivalent task (MET)-minutes per week, was recorded using the International Physical Activity Questionnaire (IPAQ) [21].

Appendicular skeletal muscle mass (kg) was quantified using bioelectrical impedance analysis (BIA, InBody 720, Biospace, Seoul, Republic of Korea) to estimate the skeletal muscle mass [22]. The BIA equipment was calibrated every morning before the start of the health examination in order to ensure the accuracy and consistency of the results. By dividing the appendicular skeletal muscle mass (kg) by the square of height (m^2^), we obtained at the skeletal muscle mass index (SMI) [23].

### 2.3. Threshold Levels of Skeletal Muscle Mass and CA 19-9

We classified the participants based on the SMI in accordance with the previous literature [24]. Participants were categorized as “normal (control)” if their SMI value was more than −1 standard deviation (SD) from the gender-specific mean for young adults (18–39 years old). Participants with a SMI value within −1 to −2 SD (−2 < SD ≤ −1) and below −2 SD (SD ≤ −2) of the gender-specific mean for young adults were categorized as ‘mild LMM’ and ‘severe LMM,’ respectively. The gender-specific cutoff values for mild and severe LMM in this study population were 7.44 and 6.78 kg/m^2^ for males and 5.48 and 4.86 kg/m^2^ for women, respectively. Therefore, the participants were grouped into three groups of ‘normal muscle mass (control)’, ‘mild LMM’, and ‘severe LMM’.

The threshold level for CA-19-9 was set as 30 U/mL. Participants with CA 19-9 ≥ 30 U/mL were defined as high CA 19-9, while those with CA 19-9 level < 30 ng/mL were defined as normal CA 19-9 level based on a prior study [25,26]. The Kangbuk Samsung Hospital Laboratory Medicine Department, which has been approved by the Korean Association of Quality Assurance for Clinical Laboratories and Korean Society of Laboratory Medicine, verified all laboratory test results [17]. 

As values of CA 19-9 were extremely skewed, CA 19-9 level was analyzed in natural logarithm form: ln (CA 19-9), as previously reported [27].

### 2.4. Statistical Analysis

The Chi-square test was used to compare the categorical variables, and one-way analysis of variance (ANOVA) was used to compare the continuous variables. Prevalence (%) of high CA 19-9 levels among the normal, mild LMM, and severe LMM groups was compared using the Chi-square test and post-hoc analysis, using Bonferroni method for correction. Furthermore, because of its positively skewed distribution, we chose the natural log-transformed (ln) CA 19-9 level, which provided the best-fitting model for analysis in which the CA 19-9 level was regarded as a continuous variable. Comparison of adjusted mean log-transformed (CA 19-9) among the groups was done using ANCOVA after adjustments for age, sex, screening center, smoking status, heavy drinker, MET minutes per week, SBP, glucose, triglyceride, ALT, creatinine, and CRP. 

Multivariable logistic regression analyses were conducted to assess the association of high CA 19-9 level with mild LMM and severe LMM. Three models with adjustment for confounding factors were used. Adjustment for each model was as follows: model 1, adjustments for age, sex, and screening center; model 2, adjustments for model 1 plus health-related behavior factors (smoking status, heavy drinker, and MET minutes per week); model 3, model 2 plus SBP, glucose, triglyceride, ALT, creatinine, and CRP. 

Odds ratio (OR) was used to calculate the risk of having mild and severe LMM compared to the normal group of participants with high CA 19-9 level, along with the calculation of the 95% confidence interval (CI). Subgroup analysis by age and sex was performed using model 3. Subgroup analyses were conducted based on the age (younger [aged <40 years], middle-aged [40–59 years], and older [≥60 years]) in both men and women for comparing the associations between CA 19-9 and LMM. For statistical analysis, two-tailed *p* value < 0.05 was considered significant. We used IBM SPSS version 26.0 (IBM Co., Armonk, NY, USA) for all statistical analysis.

## 3. Results

### 3.1. Baseline Characteristics

A total of 263,061 participants were included. The average (±SD) age was 41.0 ± 9.5 years, and the proportion of men was 50.8% (Table 1). The number of participants with normal muscle mass (control), mild LMM, and severe LMM was 226,759 (86.2%), 32,532 (12.4%), and 3770 (1.4%), respectively. The average SMI was 7.3 ± 1.1 kg/m^2^ in normal, 6.4 ± 1.0 kg/m^2^ in mild LMM, and 6.0 ± 0.8 kg/m^2^ in the severe LMM group. There were significant between-group differences in all baseline characteristics except for the screening center (*p* = 0.456) and total cholesterol (*p* = 0.931).

### 3.2. Comparison of CA 19-9 Level among Groups Classified by a Low Skeletal Muscle Mass

**Figure 2:**
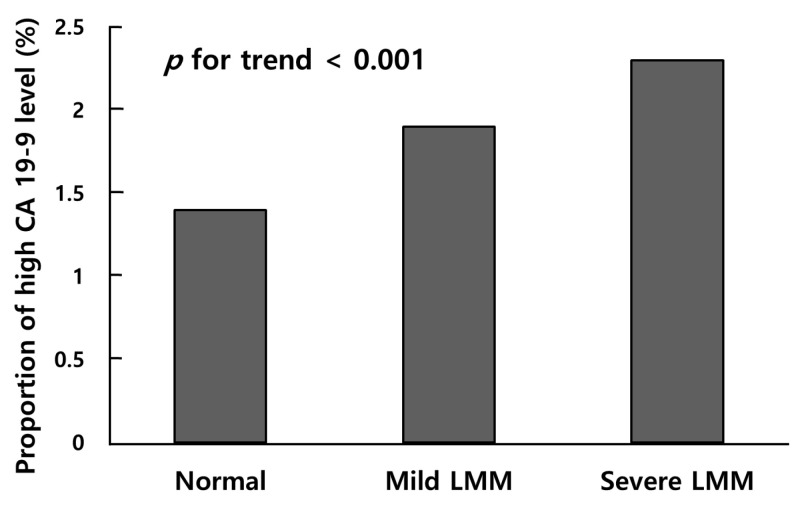
Comparison of proportion of high CA 19-9 level between normal, mild LMM, and severe LMM group. CA 19-9 levels in the groups were compared using chi-square test. *p* for trend < 0.001. CA 19-9: carbohydrate antigen 19-9; LMM: low muscle mass.

**Figure 3:**
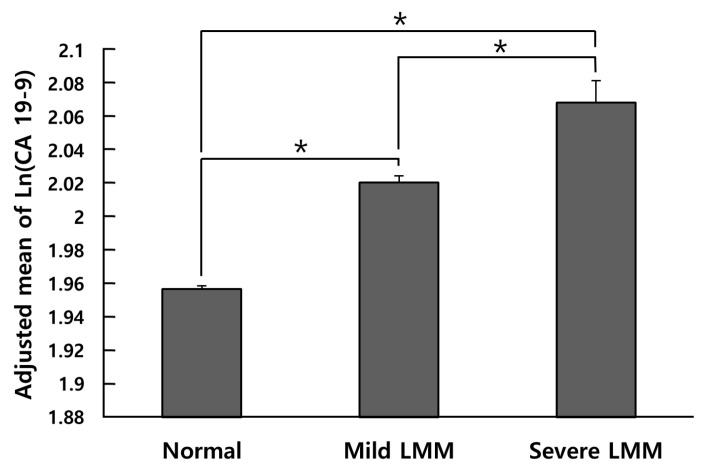
Comparison of adjusted mean of ln(CA 19-9) between normal, mild LMM, and severe LMM group. Adjusted means (±SE) of natural-log-transformed CA 19-9 levels in the groups were estimated from ANCOVA after adjustments for age, sex, screening center, smoking status, heavy drinker, MET minutes per week, SBP, glucose, triglyceride, ALT, creatinine and CRP. *: Group difference by Bonferroni post hoc *p* < 0.001. CA 19-9: carbohydrate antigen 19-9; LMM: low muscle mass; MET: metabolic equivalent task; ALT: alanine aminotransferase; CRP: *C*-reactive protein; SE: standard error.

The prevalence of high CA 19-9 level was the highest in the severe LMM group (2.3%), followed by the mild LMM (1.9%) and normal muscle mass groups (1.4%) (*p* for trend < 0.001) (Figure 2). After adjustments for possible confounding factors, including age, sex, screening center, smoking status, heavy drinker, MET minutes per week, SBP, glucose, triglyceride, ALT, creatinine, and CRP, the adjusted mean of ln (CA 19-9) was the highest in the severe LMM group, followed by the mild LMM group and those with a normal muscle mass (*p* for trend < 0.001) (Figure 3). The adjusted mean CA 19-9 value in all group comparisons showed significant differences in post hoc Bonferroni analysis (all post hoc *p* < 0.001).

### 3.3. Association of High CA 19-9 with Low Skeletal Muscle Mass

Multivariable logistic regression analyses were conducted for the association between a high CA 19-9 level and LMM (Table 2). In model 1, after adjustments for age, sex, and screening center, high CA 19-9 level was significantly associated with mild LMM (adjusted odds ratio [aOR], 1.531; 95% CI, 1.403–1.672) and severe LMM (aOR, 2.280; 95% CI, 1.835–2.835) but not with normal muscle mass (control). The second model (model 2) additionally adjusted for health-related behavior factors, such as the smoking status, heavy drinker, and MET minutes per week; aOR (95% CI) of the high CA 19-9 group was 1.530 (1.402–1.671) for having mild LMM and 2.258 (1.816–2.807) for having severe LMM, respectively. In model 3 with additional adjustment for SBP, glucose, triglyceride, ALT, creatinine, and CRP, high CA 19-9 level was independently associated with having a mild LMM (aOR, 1.677; 95% CI, 1.533–1.834) and severe LMM (aOR, 2.651; 95% CI, 2.126–3.306), respectively, compared to having normal muscle mass.

### 3.4. Subgroup Analysis by Age and Sex

Multivariate logistic regression analyses (model 3) were repeated in subgroups by age and sex. The subgroup analyses were conducted in groups stratified by age (<40, 40–59 years, ≥60) and sex (Table 3). All age groups, including the younger (aged < 40 years), middle-aged (40–59 years), and older (≥60 years) subgroups demonstrated significant associations of increased CA 19-9 with mild and severe LMM, respectively. However, there were no differences among the age-groups (*p* for interaction = 0.869). In subgroup analysis by sex, both men and women showed a significant association between high CA 19-9 level and severe LMM. Furthermore, there was a stronger association in men than in women (*p* for interaction = 0.003).

## 4. Discussion

In the present study, elevated CA 19-9 level was significantly associated with a higher prevalence of mild and severe LMM in a large group of healthy adults, even after adjustments for various possible confounding factors. Furthermore, the significant associations persisted in subgroup analyses by age and sex. Interestingly, men demonstrated a stronger association between high CA 19-9 and LMM compared to women. To the best our knowledge, this is the first study to evaluate the potential relationship between elevated CA 19-9 and low muscle mass in healthy adults without cancer.

CA 19-9 is a type of carbohydrate structure belonging to the Lewis family of blood antigens, which is synthesized in epithelial cells in the pancreas, bile duct, and other organs [8]. This biomarker is widely used for pancreatic cancer diagnosis due to its high expression in malignant conditions [10]. However, its sensitivity and positive predictive value (PPV) are relatively low, with only a PPV of 0.9% reported by Kim et al. along with 1059 false-positives, making it unsuitable for use as a screening marker [28,29]. Nevertheless, CA 19-9 is widely used as a diagnostic and screening test as it is the most validated biomarker, and it also holds significant prognostic value and provides information on operability.

Elevated CA 19-9 levels have been commonly observed in malignant diseases including cancers of the pancreas, colon and rectum, liver, gallbladder, and bile duct [8,30,31]. The underlying pathophysiological mechanism is attributed to the structurally incomplete carbohydrate determinant of CA 19-9 because of malignantly transformed epithelial cells exhibiting surface glycans. Additionally, hypoxia-resistant tumor cells that are prevalent in advanced tumors have been known to induce increased synthesis of CA 19-9 [32]. Not only malignancies but benign diseases such as cholangitis, pancreatitis, bile duct obstruction, gallstones, and chronic hepatitis have also been associated with elevated CA 19-9 levels [13,16]. The mechanism involves non-tumorous epithelial cell inflammation, block to excretion of CA 19-9, and impaired CA 19-9 metabolism. More recently, non-hepatopancreatobiliary diseases, including interstitial lung disease, urological disease, and rheumatic disease have also been associated with elevated levels of CA 19-9 [12,14,33,34,35]. In these cases, metaplastic epithelial cells in the lung tissue and uropelvic junction epithelium are positively stained for the anti-CA 19-9 antibody, but the mechanism is not fully elucidated yet.

Furthermore, even in the absence of definite causes, sustained elevation of CA 19-9 has been reported in “normal” adults. Kim et al. investigated the cause of elevated CA 19-9 levels in 192 patients who showed no signs of malignant or pancreatobiliary disease among a total of 6899 patients with elevated CA 19-9 levels. The highest incidence was found to be in hepatic disease at 32.8%, but the cause was still unknown in 23.4% patients, and the level did not normalize in 28.6% patients within three months of follow-up [15]. Lee et al. found that among 581 participants who initially had no causative disease out of a total of 58,498 individuals who visited a health inspection center, only 36.8% had a causative condition confirmed during follow-up; however, a remarkable 63.2% had no explainable disease condition, and among these participants, 23% had sustained or fluctuating high levels of CA 19-9 [16]. Therefore, there could be an unexplained portion of CA 19-9 elevation that can be attributed to physiological characteristics in the population without disease-related associations. 

Our results indicate that there may be a relationship between elevated CA 19-9 and LMM, which has not been reported to date. As the progression of malignancy is associated with cancer outcomes and muscle wasting, the independent correlation between LMM and CA 19-9 may have been overlooked. In addition, the relationship between LMM and CA 19-9 may be masked when investigating cancer patients, as malignant cells predominantly express CA 19-9. Previously, it was reported that CA 19-9 levels were significantly different between muscle gainers and muscle losers after curative pancreas resection for cancer [36]. Additionally, sarcopenia only affected the overall survival rates and not the recurrence-free survival rates, suggesting that factors other than tumor biology contributed to the mortality rates. Thus, it is possible that CA 19-9 levels may serve as an indicator of muscle gain or loss, and this affects the mortality rates without being affected by the tumor biology.

Therefore, we made speculations regarding the correlation between high CA 19-9 and LMM. Firstly, there is a correlation between insulin resistance and CA 19-9. Among non-malignant and non-pancreatobiliary tract diseases where CA 19-9 is elevated, diabetes is one of the most well-known conditions. CA 19-9 has been observed to be correlated with plasma glucose and insulin resistance [37], while insulin resistance is known to cause increased protein catabolism, expression of the FoxO family, autophagy, and decreased protein synthesis in skeletal muscle cells which are related to LMM [38,39,40,41,42,43,44]. In our study, the prevalence of diabetes was significantly higher in the severe LMM group, and even after including fasting glucose in the multivariable analysis, the correlation between LMM and CA 19-9 remained. It is possible that subclinical insulin resistance and associated subclinical pancreatic function may partially affect the correlation between LMM and these variables. In 2012, Haoyong reported that insulin deficiency is directly associated with pancreatic exocrine deficiency and release of CA 19-9 by ductal cells [45]. Additionally, they found that the level of serum CA 19-9 was higher in type 1 diabetes than in type 2 diabetes. These results also support the theory that increased serum CA 19-9 levels may be a biomarker of damaged pancreatic beta cell function.

Secondly, it has been reported that pancreatobiliary epithelial cells produce a significant amount of CA 19-9 in relation to inflammation [13]. The association of LMM with chronic inflammation is well known, with the involvement of pro-inflammatory cytokines such as interleukin-1beta, interleukin-6, and tumor necrosis factor alpha in muscle breakdown [46,47,48,49]. Therefore, it is possible that CA 19-9 increases in relation to LMM-induced chronic inflammation of pancreatobiliary epithelial cells [50].

Thirdly, sarcopenia is highly prevalent in individuals with hepatocellular dysfunction conditions, such as hepatitis, liver cirrhosis, and liver transplantation due to the crucial role of hepatic function in protein synthesis and glycogen metabolism [51]. In our study, 52,525 patients with a history of chronic liver disease and liver cirrhosis were excluded, and AST/ALT of enrolled individuals with LMM were in the normal range; however, subclinical hepatocellular hypofunction or inflammation may be associated with LMM due to decreased protein synthesis, and it could be partially represented by CA 19-9.

In the subgroup analysis by age and sex, all age subgroups showed a significant association between CA 19-9 and LMM, but there was no age effect in the multivariable analysis. However, there was a significant sex effect in male participants, with the OR of the severe LMM group for increased CA 19-9 being 3.418.

A previous study by Lee et al. on normal individuals of Korean ethnicity reported that people with older age and women showed higher CA 19-9 levels, along with diabetic individuals and those with low BMI [16]. Our study also showed that individuals with older age and women had higher mean CA 19-9 levels, and a higher ratio of individuals with high CA 19-9 levels were over 30 years old. However, our study revealed an interesting fact that despite higher baseline CA 19-9 levels in women, CA 19-9 was more sensitive as a biomarker for LMM in men. This has not been reported previously.

Sex differences in the body composition and energy balance are well known. The first explanation for this difference is related to sex differences in the body composition; women generally have a higher proportion of adipose tissue, while men tend to have more visceral adipose tissue than women, because men tend to have central fat distribution compared to peripheral fat distribution observed in women [52,53,54,55,56,57]. Therefore, an increase in adipose tissue related to LMM would likely occur more significantly in the visceral organs, including the pancreas and gall bladder in men. Consequently, the pro-inflammatory state and dysfunction of pancreatic cells due to LMM-induced increase in adipose tissue may further increase the CA 19-9 levels. Similarly, the male sex was reported as an independent risk factor for nonalcoholic fatty liver disease [58].

The second explanation is related to estrogen’s role in maintaining glucose homeostasis stress [59,60,61,62,63]. Estrogen plays a protective role against hyperglycemia in diabetic states, resulting in lower insulin resistance in women compared to men. Therefore, subclinical insulin resistance due to LMM and its correlation with CA 19-9 may be higher in men in relation to pancreatic dysfunction described earlier [64]. Finally, estrogen directly protects pancreatic beta-cell function from oxidative stress [59,60,61]. An increase in adipose tissue not only increases inflammation and insulin resistance but also oxidative stress [2]; therefore, men with LMM may receive less protection against oxidative stress in their pancreas and may have a greater likelihood of producing more CA 19-9. Further research is needed to clarify the effect of sex on this correlation. 

This study has several strengths. Firstly, it was a population-based study that reports for the first time the correlation between CA 19-9 and LMM in individuals without cancer, with a large sample size of 263,061. Secondly, the analysis was adjusted for various confounding factors using multivariate techniques, including health-behavior factors such as smoking, alcohol consumption, and physical activity quantified as MET-minutes per week that significantly influence muscle mass, as well as biochemical laboratory factors. The results suggest that CA 19-9 can be considered as a biomarker indicating LMM in individuals without cancer. Moreover, since CA 19-9 is a widely used laboratory test routinely performed during health checkups, it can be used efficiently on-site without additional medical expenses.

In the current clinical practice, LMM could be considered as a possible condition in the systematic examination process of patients with C A19-9 levels above 30 U/mL which is used in present practice. For example, if a patient’s CA 19-9 levels exceed 30 U/mL, it is crucial to prioritize the detection or monitoring the occurrence of cancer. However, once malignancy is ruled out, it is important to consider the possibility of benign conditions unrelated to cancer, such as the presence of LMM. Furthermore, the multivariate odds ratios for the occurrence of mild and severe LMM in relation to high serum CA 19-9 are comparable to or higher than those reported for lifestyle and health behavior factors in previous studies [3,65]. This highlights the necessity for caution and proactive implementation of exercise and dietary interventions.

Additionally, by demonstrating a significant association between serum CA 19-9 levels and LMM, clinicians and researchers can now consider conducting further investigations to determine the causal relationship between these two factors. Specifically, longitudinal cohort studies and research into the underlying biological mechanisms can be pursued. Additionally, incorporating CA 19-9 as a feature in the development of prediction models for sarcopenia could enhance their accuracy and predictive power.

## 5. Limitation

This study had a retrospective and cross-sectional design, which means that the results suggest a relationship rather than causation. Prospective studies in the future will be necessary to provide further clarification. Additionally, the lack of follow-up may have missed potential malignancies, pancreatitis, or other conditions that could have increased CA 19-9 levels. However, we made every effort to include only normal subjects and excluded 116,145 individuals accordingly. Furthermore, the study population consisted of only one ethnic group, thereby limiting its external validity for other ethnicities, although it provides strong internal validity for the Korean population. Finally, we did not account for the Lewis blood group phenotype, which may have interfered with our results since certain phenotypes exhibit higher CA 19-9 levels than others and may not be expressed in genotype-negative individuals [66,67]. However, this is an unavoidable limitation of this large population study using health examination data due to medical costs. Nonetheless, our sample size was large and comprised individuals of a single ethnicity, reducing the likelihood of group bias towards a specific phenotype.

## 6. Conclusions

In conclusion, our findings suggest that elevated levels of CA 19-9 are independently associated with a higher prevalence of LMM in apparently healthy adults without cancer. These results support the use of CA 19-9 as a potential biomarker for detecting the LMM status. Future research should focus on elucidating the physiological mechanisms underlying this association.

## Figures and Tables

**Table 1:** Baseline characteristics of participants classified by skeletal muscle mass.

	Total	Normal	Mild LMM	Severe LMM	* *p* Value
Number of subjects (*n*)	263,061	226,759	32,532	3770	
Age (years)	41.0 ± 9.5	41.0 ± 9.2	41.1 ± 10.8	44.2 ± 13.5	<0.001 ^‡,#^
Sex, Men (%)	50.8	49.6	56.9	73.5	<0.001
Screening center, Seoul (%)	48.3	48.3	48.5	47.4	0.456
Height (cm)	167.3 ± 8.6	167.6 ± 8.6	165.2 ± 8.1	164.3 ± 7.8	<0.001 ^†,‡,#^
BMI (kg/m^2^)	23.5 ± 3.5	24.0 ± 3.4	20.7 ± 2.2	19.4 ± 2.2	<0.001 ^†,‡,#^
Appendicular skeletal muscle mass (kg)	20.2 ± 5.0	20.7 ± 4.9	17.6 ± 4.1	16.5 ± 3.4	<0.001 ^†,‡,#^
SMI (kg/m^2^)	7.1 ± 1.1	7.3 ± 1.1	6.4 ± 1.0	6.0 ± 0.8	<0.001 ^†,‡,#^
Current smoker (%)	14.8	14.5	15.8	21.8	<0.001
Heavy drinking ^a^ (%)	15.8	15.9	15.0	18.1	<0.001
MET minutes per week (min/wk)	1584.0 ± 3037.8	1616.9 ± 3068.9	1374.2 ± 2774.1	1415.9 ± 3253.3	<0.001 ^†,‡^
Systolic blood pressure (mmHg)	109.6 ± 12.7	110.1 ± 12.7	106.5 ± 12.0	107.6 ± 13.1	<0.001 ^†,‡,#^
Diastolic blood pressure (mmHg)	70.4 ± 9.7	70.5 ± 9.8	69.2 ± 9.3	70.2 ± 9.4	<0.001 ^†,#^
Hypertension (%)	8.5	8.6	7.6	10.5	<0.001
Diabetes mellitus (%)	2.4	2.4	2.7	5.1	<0.001
Insulin (uIU/mL)	7.1 ± 4.8	7.3 ± 4.9	5.7 ± 3.3	5.1 ± 3.1	<0.001 ^†,‡,#^
HOMA-IR	1.7 ± 1.4	1.8 ± 1.4	1.4 ± 0.9	1.3 ± 0.9	<0.001 ^†,‡,#^
Glucose (mg/dL)	96.7 ± 15.2	96.9 ± 15.1	95.4 ± 15.5	96.9 ± 20.0	<0.001 ^†,#^
Triglycerides (mg/dL)	112.9 ± 78.6	114.7 ± 80.6	101.4 ± 63.0	102.4 ± 65.1	<0.001 ^†,‡^
Total cholesterol (mg/dL)	190.4 ± 33.9	190.4 ± 33.9	190.4 ± 33.9	190.6 ± 35.6	0.931
LDL-C (mg/dL)	125.6 ± 33.1	125.8 ± 33.0	124.3 ± 33.2	123.8 ± 34.4	<0.001 ^†,‡^
HDL-C (mg/dL)	60.8 ± 16.2	60.3 ± 16.1	63.9 ± 16.3	64.1 ± 17.1	<0.001 ^†,‡^
AST (IU/L)	22.2 ± 14.7	22.2 ± 14.8	21.6 ± 13.1	23.6 ± 21.1	<0.001 ^†,‡,#^
ALT (IU/L)	23.6 ± 19.9	23.7 ± 20.3	20.9 ± 16.8	21.8 ± 18.3	<0.001 ^†,‡,#^
Total bilirubin (mg/dL)	0.78 ± 0.36	0.78 ± 0.36	0.80 ± 0.37	0.83 ± 0.38	<0.001 ^†,‡,#^
γ-GTP (IU/L)	30.7 ± 39.5	30.8 ± 38.2	29.6 ± 41.6	37.2 ± 77.8	<0.001 ^†,‡,#^
Alk-phosphatase (IU/L)	59.4 ± 17.1	59.1 ± 17.0	60.9 ± 17.5	66.5 ± 21.1	<0.001 ^†,‡,#^
Creatinine (mg/dL)	0.82 ± 0.22	0.82 ± 0.22	0.81 ± 0.19	0.83 ± 0.24	<0.001^†,‡,#^
CRP (mg/dL)	0.11 ± 0.30	0.12 ± 0.29	0.10 ± 0.32	0.13 ± 0.43	<0.001 ^†,‡,#^
CA 19-9 (U/mL)	9.2 ± 10.2	9.2 ± 10.0	9.6 ± 8.6	10.4 ± 24.3	<0.001 ^†,‡,#^

Data are presented as mean ± SD, median (IQR), or percentage. * *p* values for the difference between groups in continuous variables by one-way ANOVA or in categorical variables by χ2 test. After the one-way ANOVA, group comparisons were conducted using the Bonferroni post hoc analysis. ^†^: Bonferroni post hoc *p* < 0.05 for group comparison of normal vs. mild LMM. ^‡^: Bonferroni post hoc *p* < 0.05 for group comparison of normal vs. severe LMM. ^#^: Bonferroni post hoc *p* < 0.05 for group comparison of mild LMM vs. severe LMM. ^a^ >20 g/day. CA 19-9: carbohydrate antigen 19-9; BMI: body mass index; MET: metabolic equivalent task; HOMA-IR: homeostatic model assessment for insulin resistance; LDL-C: low-density lipoprotein cholesterol; HDL-C: high-density lipoprotein cholesterol; ALT: alanine aminotransferase; AST: aspartate aminotransferase; CRP: *C*-reactive protein; LMM: low muscle mass; SMI: skeletal muscle mass index. SMI (kg/m^2^) = appendicular skeletal muscle mass (kg)/height (m)^2^.

**Table 2:** Multivariate regression analyses showing association of increased CA 19-9 with LMM.

	Mild LMM, OR (95% CI)	Severe LMM, OR (95% CI)
Model 1		
Normal (<30 U/mL)	[1] (reference)	[1] (reference)
High CA 19-9 level (≥30 U/mL)	1.531 (1.403–1.672)	2.280 (1.835–2.835)
Model 2		
Normal (<30 U/mL)	[1] (reference)	[1] (reference)
High CA 19-9 level (≥30 U/mL)	1.530 (1.402–1.671)	2.258 (1.816–2.807)
Model 3		
Normal (<30 U/mL)	[1] (reference)	[1] (reference)
High CA 19-9 level (≥30 U/mL)	1.677 (1.533–1.834)	2.651 (2.126–3.306)

ORs were calculated as the risks of having mild, low, or severely low skeletal muscle mass according to the presence of high CA 19-9 level. Model 1: adjusted for age, sex, screening center. Model 2: adjusted for age, sex, screening center, smoking status, heavy drinker, and MET minutes per week. Model 3: adjusted for age, sex, screening center, smoking status, heavy drinker, MET minutes per week, SBP, glucose, triglyceride, ALT, creatinine, and CRP. CA 19-9: carbohydrate antigen 19-9; LMM: low muscle mass; MET: metabolic equivalent task; SBP: systolic blood pressure; ALT: alanine aminotransferase; CRP: *C*-reactive protein; CI: confidence interval; OR: odds ratio.

**Table 3:** Subgroup analyses for age and sex for associations of increased CA 19-9 with LMM.

	Mild LMM, OR (95% CI)	Severe LMM, OR (95% CI)	*p* for Interaction
Age			0.869
<40 (*n* = 134,353)	1.439 (1.274–1.626)	2.056 (1.463–2.890)	
40~59 (*n* = 117,388)	1.753 (1.506–2.041)	2.397 (1.580–3.635)	
≥60 (*n* = 11,320)	1.415 (1.066–1.880)	1.998 (1.284–3.111)	
Sex			0.003
Men (*n* = 133,685)	1.939 (1.590–2.364)	3.418 (2.452–4.764)	
Women (*n* = 129,376)	1.583 (1.431–1.752)	2.117 (1.564–2.866)	

Adjusted ORs were calculated as the risks of having mild and severe low skeletal muscle mass (LMM) according to the presence of high CA 19-9 level in each subgroup after adjustments for age, sex, screening center, smoking status, heavy drinker, MET minutes per week, SBP, glucose, triglyceride, ALT, creatinine, and CRP. CA 19-9: carbohydrate antigen 19-9; LMM: low muscle mass; MET: metabolic equivalent task; SBP: systolic blood pressure; ALT: alanine aminotransferase; CRP: *C*-reactive protein; CI: confidence interval; OR: odds ratio.

## Data Availability

Data can be obtained from corresponding author upon reasonable request.
